# Over 300 Radiation Caries Papers: Reflections From the Rearview Mirror

**DOI:** 10.3389/froh.2022.961594

**Published:** 2022-07-14

**Authors:** Caique Mariano Pedroso, Cesar Augusto Migliorati, Joel B. Epstein, Ana Carolina Prado Ribeiro, Thaís Bianca Brandão, Márcio Ajudarte Lopes, Mário Fernando de Goes, Alan Roger Santos-Silva

**Affiliations:** ^1^Department of Oral Diagnosis, Piracicaba Dental School, University of Campinas (UNICAMP), Campinas, Brazil; ^2^College of Dentistry, University of Florida, Gainesville, FL, United States; ^3^Cedars-Sinai Medical Center, Samuel Oschin Comprehensive Cancer Institute, Los Angeles, CA, United States; ^4^City of Hope Comprehensive Cancer Center, Duarte, CA, United States; ^5^Dental Oncology Service, Instituto do Câncer do Estado de São Paulo (ICESP), São Paulo, Brazil

**Keywords:** radiation caries, radiotherapy, radiation, dental caries, dental demineralization, head and neck cancer

## Abstract

Radiation caries (RC) is an aggressive oral toxicity in head and neck cancer survivors, which develops 6 to 12 months after head and neck radiotherapy. It initially affects the tooth cervical/incisal surfaces, and if not promptly diagnosed/managed, progresses to dental crown amputation and risk of osteoradionecrosis. It results from a multidimensional cluster of treatment-induced oral symptoms, including hyposalivation, dietary changes, and oral hygiene impairment. Although recognized as a frequent complication of radiotherapy and extensively assessed by a myriad of retrospective, *in vitro*, and *in situ* studies, RC patients are still orphans of clinically validated methods for risk prediction, prevention, and treatment of early lesions. This review provides a historical overview of science-based concepts regarding RC pathogenesis and treatment, emphasizing the growing demand for interventional clinical studies (randomized trials).

## Introduction

Head and neck cancer (HNC) represent 6% of all malignancies affecting the world population, with over 500,000 new cases worldwide per year. More than half of the patients are diagnosed in advanced stage of disease, leading to the need for multimodal treatment including surgery followed by radiotherapy, chemoradiotherapy, and, more recently, molecular targeted therapy (immunotherapy) for advanced/recurrent/metastatic disease [1]. In this context, head and neck radiotherapy (HNRT) is a locoregional therapy that involves radiation to treat the primary tumor, and regional lymphatic drainage. Although fractionation is performed, acute and late oral complications occur in virtually all patients during and after treatment, including oral mucositis, hyposalivation, sensory changes (mucosal pain, dysgeusia), dysphagia, trismus, radiation caries (RC), and osteoradionecrosis ORN [2].

RC is a complex chronic oral complication of cancer therapy that affects up to 30% of patients within 12 months following the conclusion of HNRT [3], and risk continues indefinitely. The indirect effects of HNRT, validated by the identification of HNRT-specific cluster of symptoms, are the most accepted hypothesis for RC onset and progression [4]. Additionally, poor oral health status, lack of access to dental care before HNRT, primary oral care during and post-radiation treatment, HNRT plans and dosimetric parameters due to tumor location and stage of disease are also some of the well-recognized risk factors for RC development [5].

Despite being well recognized as an oral complication in HNC patients, RC still poses a clinical challenge in terms of risk prediction, clinically validated protocols for prevention, early diagnosis strategies, and optimal treatment interventions. These challenges negatively impact the quality of life of HNC survivors, leading to generalized tooth destruction, loss of masticatory efficiency, persistent chronic oral infections, pain, increased risk of ORN and may impact speech, diet, and esthetics [5, 6]. This review focuses on a historical assessment of RC knowledge as well as on emerging concepts regarding its management.

## Methods

To provide a focused investigation concerning RC outcomes, searches were performed in PubMed/Medline, Scopus, Embase, and Web of Science (Supplementary Table 1). Moreover, a search was performed in the Index-Catalog of the Library of the Surgeon-General's Office (US National Library of Medicine), and Medical Heritage Library, both historical research tools concerning RC. The reference lists within selected articles were manually assessed for additional studies that might have been missed during the initial search. The electronic search was performed with using following the keywords: “radiation caries” OR “radiation-related caries” OR “radiation-related dental caries” OR “radiation dental caries” OR “radiation-induced dental caries” OR “post-radiation caries”. The search was not limited by year limitations and language restrictions. We include all types of primary and secondary studies that comprised RC concepts, diagnostic and clinical features, prevention, pathogenesis, risk factors, prevention strategies, and treatment. Exclusion criteria were: (1) abstracts, book chapters, editorials, letters to the Editor, notes, commentaries, or images; (2) studies that were not associated with RC outcome; (3) studies that included non-ionizing radiation; (4) studies that assessed dental care pre-HNRT; (5) studies that assessed other oral complication of HNRT than RC; (6) abstract or full-text not accessible. EndNote^®^ (Clarivate Analytics, Philadelphia, USA) and Rayyan software were used for reports screening, exclusion of duplicates, and registration of reason for exclusion (Supplementary Table 2).

## Overview

### Definitions and the “History” of RC

**Figure 1:**
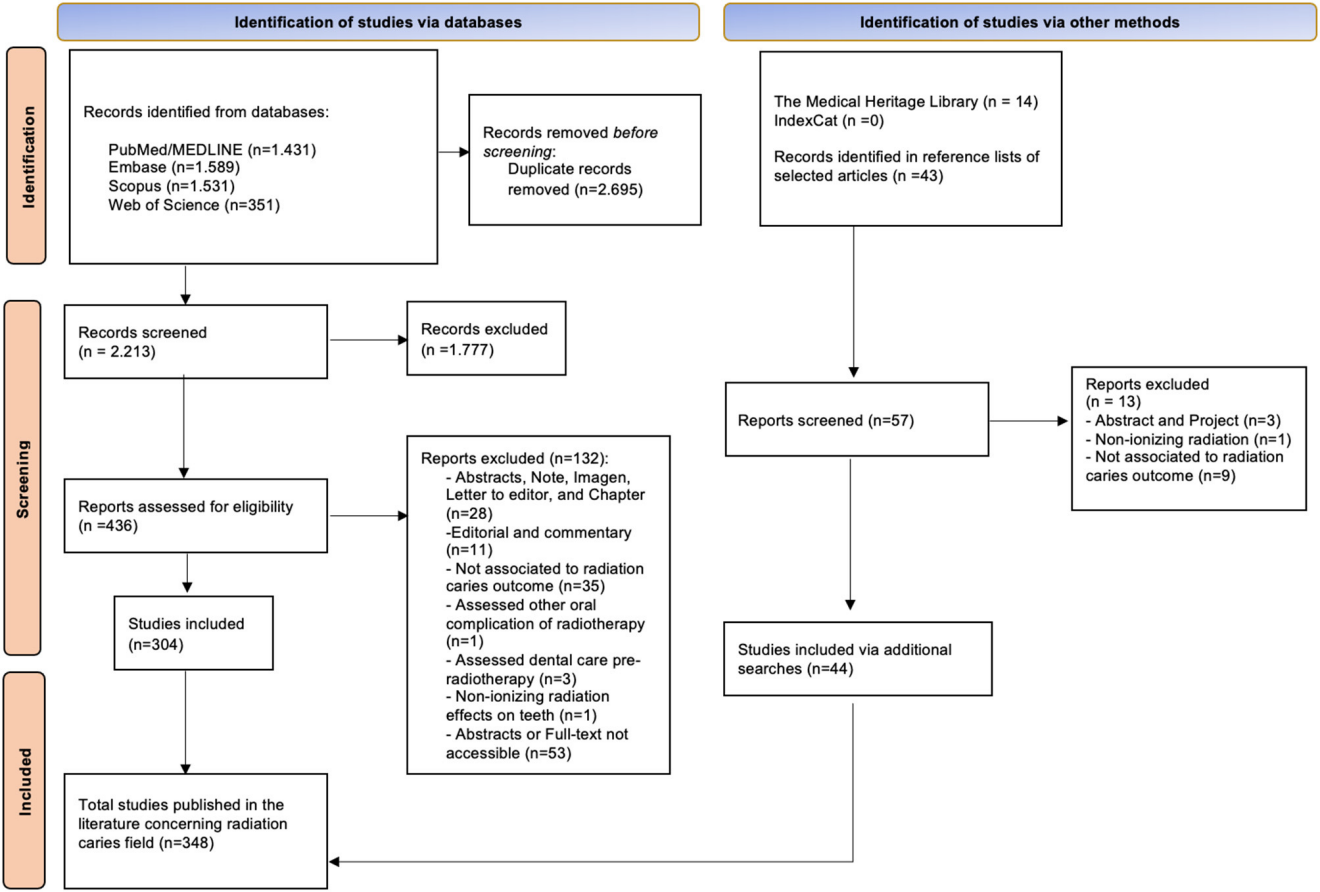
Flowchart describing literature searches.

Based on the current literature, more than three hundred articles related to RC outcomes were published over the years (Figure 1). Most of the included articles were preclinical studies (32%) followed by narrative reviews (30%), cohort studies (12%), and clinical trials (3%) (Supplementary Table 3). Over 82 years since the first report, the evidence levels of studies remain low, which contribute to the lack of well-designed clinical protocols for RC diagnosis and treatment.

The term “radiation caries” describes rampant caries following HNRT [7]. Additionally, another term described as “radiation-related caries” (RRC) has been used to refer mainly to caries associated with indirect effects of HNRT [5]. In the first half of the 20th century, RC was initially reported as an aggressive type of tooth decay with peculiar features that affect HNC patients after an oncologic treatment setting. In the early 1940s, RC lesions were found in the cervical, incisal areas and cusp tips that can lead to dental crown amputation [8] and dental abscess formation. Long-standing RC definitions concepts reported by several authors remain similar in contemporary times, especially regarding the main areas of teeth affected and fast progression patterns. The main areas of teeth affected by RC lesions are the cervical areas surrounding teeth and the lingual surfaces of the anterior mandibular teeth [5] and often extend to involve the entire dentition. Initial stage features of dental demineralization have been described over time, facilitating the recognition of the lesions.

### RC Diagnosis

Recognition of RC, to date, has no clinically validated diagnostic criteria or methods that consider classifying RC according to clinical patterns. The RC clinical presentation differs from conventional caries (Table 1). Dental indexes have been developed to help clinicians in RC diagnosis [9, 10]; however, several limitations in these indexes are observed, which cannot be clinically representative. The ICDAS and Post-radiation dental index (PRDI) scores, methods utilized for decay diagnosis, are not practical for RC use because both methods do not consider clinical progression patterns [11], which represents key clinical patterns.

**Table 1:** Difference between clinical conditions of conventional and radiation caries.

**Conventional active caries**	**Radiation caries**
**Clinical appearance**	
Frosty/rough appearance of the whitish enamel surface	Brownish pigmentation on smooth surfaces
Microcavities on pits and fissures or white-spots lesions on the smooth surface	Enamel craze lines
White-brown discoloration in the enamel	Enamel delamination
Broken enamel surface and soft dentin	Crown amputation

**Figure 2:**
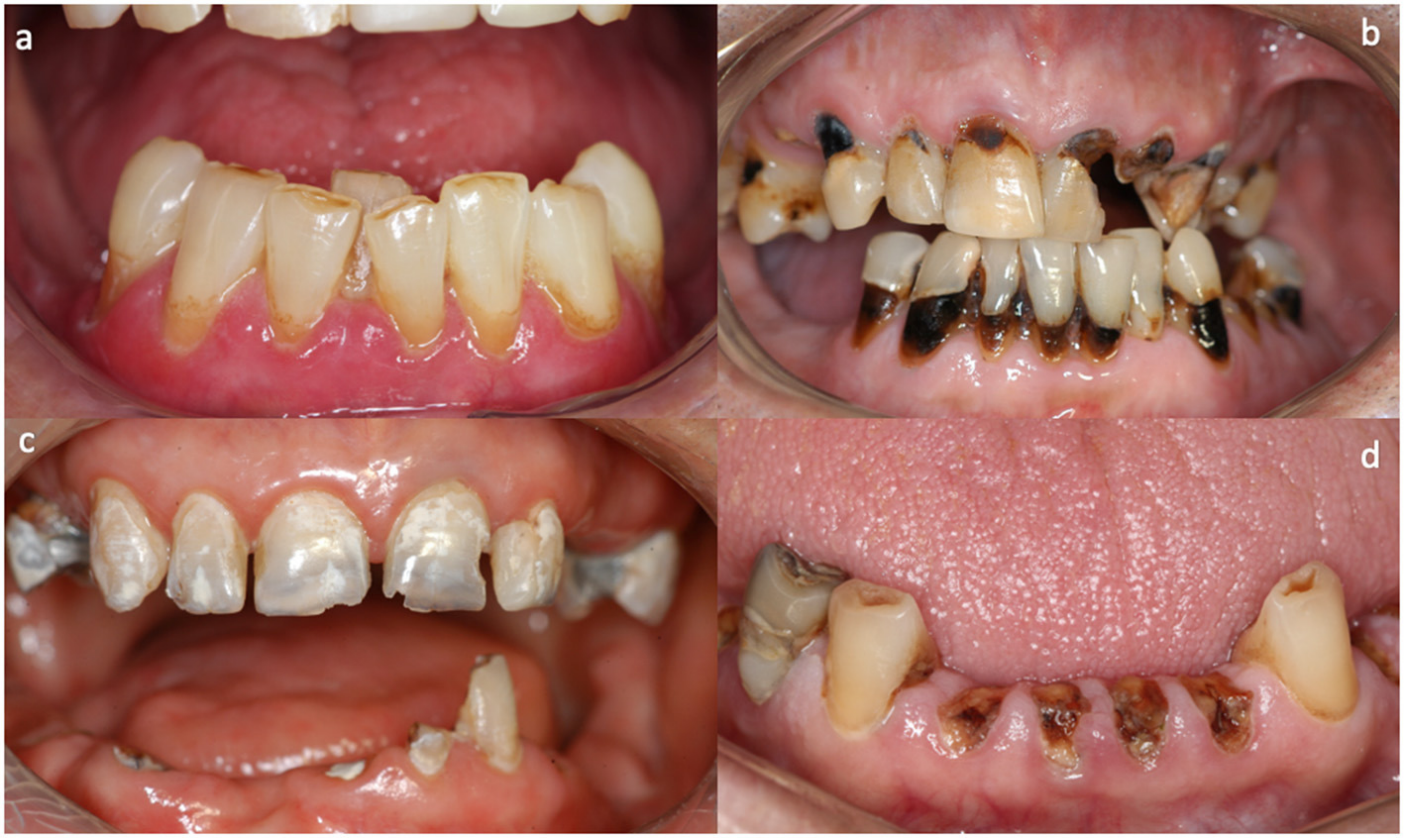
Radiation caries clinical stages. **(A,B)** Representation of incipient radiation caries with presence of superficial enamel changes with brownish pigmentation on the smooth surfaces. **(C)** Demineralization and delamination enamel spots representing the second stage. **(D)** Crown amputation is the last stage of radiation caries with progressively faster pattern.

With the lack of a systematic method for RC diagnosis, Palmier et al. [11] proposed a clinical guide to diagnosis, management, and treatment according to the clinical stage [5]. In the initial stage, RC lesions usually start with superficial enamel changes with demineralization, leading to brown/blackish pigmentation on the smooth surfaces of teeth (Figures 2A,B). Furthermore, enamel craze lines may be observed in the early stage that tend to extend from the cervical to the incisal area (Figure 2B). In the second stage, the clinical features are represented by minor demineralized spots and enamel delamination areas (Figure 2C). Posteriorly, this delamination tends to advance with extensive areas, leading to crown amputation (Figure 2D) [5]. The recognition of RC clinical features, especially in the early stages, impacts a favorable prognosis in dental restorative treatment.

Recently, with the advance in machine learning use, artificial intelligence (AI) has enabled computers to perform diagnoses and predict RC in HNC patients [12]. The use of a clinical data setting, clinical imaging, or panoramic radiography can be utilized to train AI models to predict before HNRT whether HNC patients will develop RC after treatment. Based on clinical images, AI may become an adjunct to predict clinical features of RC risk, in which the dental status before HNRT could be directly associated with the risk of developing RC.

### Pathogenesis

RC pathogenesis has been classified according to the direct and indirect effects of HNRT. The indirect effects, recently named as a “cluster of oral symptoms”, are pivotal events for the initiation of RC that leads to alterations in teeth structure. The theory of symptoms clustered is represented by hyposalivation, a highly cariogenic diet, inadequate oral hygiene, oral microbial shift, and lower pH value [4]. Recent studies reported that a decrease in oral salivary pH values causes a loss of saliva buffer capacity and biofilm accumulation that increases the cariogenic oral microbiota [13, 14]. Furthermore, alterations in the oral microbiome are a variable that may explain dental caries after radiotherapy treatment, in which the population of Streptococcus *mutans* species tends to increase 6 months after HNRT and lead to demineralization of the dental structure [15]. *In vitro* studies have reported that hotspot mutations in Streptococcus *mutans* caused by radiation doses might be among the reasons for radiation caries [16].

The hypothesis of direct effects of radiation remains unclear once this is divergent between preclinical studies that investigate RC as an outcome. Several *in vitro* studies reported that direct radiation might alter the chemical and biochemical composition of the teeth [17–19]. A decrease in enamel microhardness has been reported, which clinically might be represented as enamel craze lines and enamel delamination [20, 21]. Additionally, chemical elements (not clearly identified yet) that compose the enamel and dentin structure tend to decrease after radiation and cause teeth demineralization [21]. Although the literature suggests that direct radiation may cause morphological changes in dentition, preclinical studies have heterogeneity in the way their methodology, dosimetric parameters, and outcomes are analyzed. The heterogeneity observed implies conflicting results between preclinical studies and, to date, cannot be assumed to be a valid hypothesis exclusively related to RC pathogenesis.

In terms of pathogenesis, it is relevant to highlight that RC development at early stages is frankly asymptomatic and even when progressive may not cause pulp necrosis. A systematic review revealed that HNRT did not induce pulp necrosis [22], and recent *ex vivo* studies hypothetically affirmed that the direct effect of radiation did not impair the microvasculature or innervation of the dentin-pulp complex permanently after ionizing radiation [23, 24], which was validated by clinical studies [25, 26]. This silent progression of RC with absence of pain, particularly at early phases is an important feature to be elucidated, to better understand the mechanisms and pulpal effects caused by HNRT on the dentin-pulpal complex. Therefore, the so-called “inside out” effects of ionizing radiation on the dentin-pulp complex might impact on pulp vitality but still underexplored [26].

### Risk Prediction

RC risk predictions are generally related to dental status and HNRT dosimetric parameters. The presence of tooth decay before HNRT, due to poor oral health, smoking habits [27] and dietary changes increases the risk of RC development and tooth extraction. The extractions of teeth with RC may be necessary in advanced cases, and most of the teeth extracted after HNRT are due to RC progression and represent a significant risk factor for ORN [28]. When RC-related tooth extraction is recommended, it should involve minor trauma with minimal flap surgery whenever possible [5]. If extraction can be avoided, with restoration placed in tooth and on residual root tip, and endodontics of residual roots if needed, risk of ORN is reduced.

A previous cohort study reported that an average of eight teeth of HNC patients are decayed before the start of cancer therapy, and about 41% of teeth are a potential candidate to be extracted before or after HNRT [29]. The dental status assessed before starting HNRT is necessary to predict each patient's risk and provide urgent dental treatment. To predict lesions of RC, a first study in the literature utilized the artificial neural network based on panoramic radiographs as an option for RC detection, which showed an accuracy of 99.2% [12]. This methodology shows that further studies can be helpful in RC detection and prediction to improve the dental care of HNC patients. This could guide the selection of dosimetric parameters utilized in the oncologic setting. Dosimetric dental maps have contributed to assessing the prediction of doses in individual teeth and helped improve clinical workflow efficiency [30]. Therefore, radiation oncologists should recognize the challenges faced by dental oncologists in HNC patients adapting their radiation fields to minimize dental and salivary glands exposure [29].

High radiation doses may negatively impact HNC patient dentition with a significant risk of ORN, in which the teeth tend to be extracted early when they receive radiation doses >60 Gy [28]. It is worth mentioning that the increase in tooth loss due to RC is not a prediction related to radiation doses applied directly to the surface of the teeth and includes the significant impact of hyposalivation upon the dentition. Radiation and other events, such as salivary changes and oral microbiome shifts related to saliva functions, are related to RC pathogenesis [28]. Clinicians should consider these events when creating prevention strategies and decreasing the progression of RC lesions.

### RC Prevention Strategies

Prevention is the key to decreasing the risks of RC. HNC patients must be constantly educated about the importance of using fluoride, dental hygiene maintenance, and management of hyposalivation pre-, during, and post-HNRT [5], in addition remineralization product use. The use of intraoral positioning appliances (stents) during HNRT which when effective in reducing direct RT salivary gland exposure can reduce salivary changes and reduce caries risk [31]. Although caries rates in HNC patients were not associated simply with salivary flow reduction, the presence of residual saliva is crucial in RC prevention together with other remineralizing products. Some remineralizing products can support tooth remineralization and caries control, such as casein phosphate polypeptide-amorphous calcium phosphate in toothpaste and resin-modified glass ionomer cement [32]. In addition, fluoride and chlorhexidine varnishes should be considered once the demineralization/remineralization process protects the tooth surfaces against the oral acid environment.

Furthermore, HNC patients must be instructed about oral hygiene and RC concepts before the start of HNRT and this must be reinforced during the follow-up appointments. Previous knowledge of RC concepts by HNC patients before treatment is an excellent step in RC prevention. HNC patients' awareness of the effect of HNRT toxicities is directly related to their lower education level. Evidence shows that 75% of HNC patients do not know about the RC concepts impacting prevention [33]. HNC patient survivors who have not been assessed and provided with oral care before HNRT tend to have a high score of teeth decay after 1 year of treatment. Moreover, the greatest tooth failure occurred in HNC patients who were noncompliant before treatment and during the follow-up [27] which should be carefully considered in pretreatment dental care and prevention protocol.

Although pre-HNRT dental prevention has been performed, there is a risk of substantial tooth failure occurring within 2 years after treatment [28]. Therefore, patients should be provided with written and verbal instructions regarding oral care before, during, and after treatment, and prevention must be reinforced. They should be educated about risk factors to decrease the impact of the cluster of oral symptoms on the dentition. In addition to written and verbal orientations, educational videos have been demonstrated to be a useful audiovisual tool for understanding radiation-related side effects [34]. The audiovisual tool may be periodically presented during dental consultation, validating it as a prevention strategy to decrease the harmful dental conditions found in HNC patients.

### Treatment of Dental Damage

When there is the presence of harmful clinical conditions in dentition after HNRT, the management of RC becomes a challenge for dental clinicians. Aggressive RC progression, represented by irregular delamination of enamel and crown amputation, makes difficult the use of routine dental restoration techniques to acquire better mechanical retention [5]. Dental adhesive materials such as resin-modified glass ionomer cement (RMGIC), composite resins (CR), and glass ionomer cement (GIC) are dental materials that are often used in the treatment of structural damage of RC worldwide. The literature supports this indication, emphasizing the fact that these materials improve mechanical properties. Nevertheless, GIC and RMGIC longevity are affected by radiation-related hyposalivation because they are soluble materials, leading to higher restorative failure rates [5]. However, it is critical that the demineralization and caries process be addressed, or new and progressive dental damage will recur even after structural repair of tooth structure.

To date, few clinical trials have assessed the long-term efficiency of dental restoration with these dental adhesive materials. The chance of restoration failure is highly possible due to the deterioration aspects of irradiated teeth. A recent study reported that the rates of dental restoration failure are more significant in RMGIC and GIC than in CR, and therefore, the authors suggest the use of CR associated with fluoride gel compliance for restoring class V lesions after HNRT [35]. Furthermore, the mechanical behavior of composite resins and adhesive systems seem to be the best alternative to RC treatment [36].

The adhesive restorative treatment protocol may be divided into two steps. The first step is to expose the RC tissue, mainly when root decay occurs, and perform enamel bezel and cavity cleaning. Second, after preparing the teeth before receiving the restorative treatment, selective enamel conditioning with 37% phosphoric acid and dental conditioning with an adhesive system are performed following tooth restoration with resin composite and polishing with sanding disks. Cervical adaptation of the restoration and fully covered smooth vestibular surfaces are essential without the presence of enamel craze lines [5]. Regular dental follow-up should be performed every 3 months to reinforce dental education, perform the management of RC early lesions, and treat advanced cases [37].

### Potential Future Development in RC Research

Further studies should consider assessing clinically validated methods to standardize RC diagnosis and treatment. A specific clinical classification system would help clinicians have better success rates when using adhesive restorative protocols and decrease treatment failures. Furthermore, protocols for management should be better described and evidence-based. Primary prevention strategies should focus on the weakening of the clustering of oral symptoms and be based on oral care improvement, dosimetric studies, microbiological surveillance, and fluoride supplementation. The early RC clinical signs screening supported by AI algorithms are promising tools to help clinicians toward secondary prevention and personalized treatment.

## Conclusions

The lack of a validated clinical system for RC prediction and diagnosis, and asymptomatic early RC involvement contributes to late detection, foments inefficient preventive interventions, and ultimately limits the longevity of restorative adhesive protocols. Prevention and intervention must be comprehensive and include pre-RT treatment dental intervention and prevention, with ongoing expert care addressing all components of RC risk. Therefore, acknowledging RC risk predictors before and during HNRT is paramount when designing future clinical studies for head and neck cancer survivors. It is important to reflect on the fact that most of the 300 studies in this context have been focused on the pathogenesis of RC and conducted through pre-clinical analyses. It is time to focus on randomized clinical trials for a better understanding of the apparent asymptomatic clinical progression of CR, as well as the development of more effective methods of prevention and restorative treatment. Meanwhile, daily fluoride supplementation, dietary counseling, oral hygiene support, and *ad infinitum* post-HNRT dental follow-up are highly recommended for the dental management of head and neck cancer survivors.

## Author Contributions

CP and AS-S wrote the manuscript. ML, MG, and CM reviewed the manuscript. AS-S, AR, and TB designed and contextualized the study idea and reviewed this paper. All authors contributed to the article and approved the submitted version.

## Funding

This study received funds to cover open access publication fees from Programa de Pós-Graduação em Materiais Dentários da Faculdade de Odontologia de Piracicaba, Universidade Estadual de Campinas, CAPES-PROEX, Brazil.

## Conflict of Interest

The authors declare that the research was conducted in the absence of any commercial or financial relationships that could be construed as a potential conflict of interest.

## Publisher's Note

All claims expressed in this article are solely those of the authors and do not necessarily represent those of their affiliated organizations, or those of the publisher, the editors and the reviewers. Any product that may be evaluated in this article, or claim that may be made by its manufacturer, is not guaranteed or endorsed by the publisher.
