# Supplementary material for: Over 300 Radiation Caries Papers: Reflections From the Rearview Mirror
Source: Front Oral Health. 2022 Jul 14;3:961594. doi: 10.3389/froh.2022.961594 (PMC9330023; doi:10.3389/froh.2022.961594)
Supplement: Supplementary file 1 [file Table_1.docx]

| **Database** | **Searches**  **March 22, 2021** | **References** |
| --- | --- | --- |
| **PubMed/MEDLINE** | "radiation caries"[All Fields] OR "radiation-related caries"[All Fields] OR "radiation-related dental caries"[All Fields] OR "radiation dental caries"[All Fields] OR (("radiation"[All Fields] OR "radiotherapy"[All Fields]) AND ("dental caries"[MeSH Terms] OR ("dental"[All Fields] AND "caries"[All Fields]) OR "dental caries"[All Fields])) OR "post-radiation caries"[All Fields] | 1.431 |
| **Scopus** | TITLE-ABS-KEY ( "radiation caries" OR "radiation-related caries" OR "radiation-related dental caries" OR "radiation dental caries" OR ( "radiation" OR "radiotherapy" ) AND ( "dental caries" ) OR "post-radiation caries" ) | 1.531 |
| **Embase** | (‘radiation caries' OR 'radiation-related caries' OR 'radiation-related dental caries' OR 'radiation dental caries' OR (('radiation' OR 'radiotherapy') AND 'dental caries') OR 'post-radiation caries’) | 1.589 |
| **Web of Science** | ALL=("radiation caries" OR "radiation-related caries" OR "radiation-related dental caries" OR "radiation dental caries" OR (("radiation" OR "radiotherapy") AND "dental caries") OR "post-radiation caries") | 351 |
| **IndexCat** | (“radiation caries”) | 0 |
| **Medical Heritage Library** | (“radiation caries”) | 14 |

**Supplementary Table 1:** Search strategies in the databases and grey literature.
