# Supplementary material for: Over 300 Radiation Caries Papers: Reflections From the Rearview Mirror
Source: Front Oral Health. 2022 Jul 14;3:961594. doi: 10.3389/froh.2022.961594 (PMC9330023; doi:10.3389/froh.2022.961594)
Supplement: Supplementary file 2 [file Table_2.docx]

| **Supplementary table 2**. Excluded articles in alphabetically ordered and reasons for exclusion | | | | |
| --- | --- | --- | --- | --- |
| **Author** | **Title** | **Year** | **Journal** | **Exclusion Criteria** |
| - | Oral care for cancer patients | 2020 | Journal of the Dental American Association | Not associated to radiation caries outcome |
| - | Oral mucositis due to cancer treatments: Orodental hygiene and ice cubes | 2008 | Prescrire International | Assessed other oral complication of radiotherapy |
| - | Mechanism and causes of tooth decay | 2015 | Prescrire International | Not associated to radiation caries outcome |
| - | Pretreatment assessment and dental management of patients with nasopharyngeal carcinoma |  |  | Assessed dental care pre-radiotherapy |
| Abed H et al. | Dentists' perspectives on denture provision after radiotherapy for head and neck cancer: an exploratory study using the theoretical domains framework | 2021 | British Dental Journal | Not associated to radiation caries outcome |
| Al-Dakkak I | The association between cancer treatments and oral diseases | 2011 | Evidence-based dentistry | Commentary |
| Al-Jobair, A | Dental management of acute lymphoblastic leukemia: A case report | 2015 | Head and Neck | Abstract |
| Al-Nawas B, Grötz KA | Prospective study of the long term change of the oral flora after radiation therapy | 2006 | Support Care Cancer | Not associated to radiation caries outcome |
| Alajbeg I | Strategies for prevention and treatment of head and neck radiotherapy complications | 2012 | Support Care Cancer | Abstract |
| Ali MHM, Nurelhuda NM | Oral health status and its determinants in children with leukaemia at the Radiation and Isotope Center Khartoum, Khartoum State, Sudan | 2019 | Sudan Journal Paediatric | Not associated to radiation caries outcome |
| Alikhasi M et al. | Step-by-step full mouth rehabilitation of a nasopharyngeal carcinoma patient with tooth and implant-supported prostheses: A clinical report | 2011 | Contemp Clin Dent | Not associated to radiation caries outcome |
| Almendra Mattos RM, de Mendonça RMH, Dos Santos Aguiar S | Adherence to dental treatment reduces oral complications related to cancer treatment in pediatric and adolescent patients | 2020 | Support Care Cancer | Not associated to radiation caries outcome |
| Andersson T et al. | Odontological problems in radiotherapy | 1975 | Sven Tandlak Tidskr. | Not accessible |
| Andrews N | Dental implications and management of head and neck radiotherapy patients | 2000 | Annals of the Royal Australasian College of Dental Surgeons | Abstract |
| Araújo Andrade S | Is there evidence that radiotherapy for head and neck cancer influences the incidence of dental caries? | 2020 | Evidence-based dentistry | Commentary |
| Babu NA et al. | Dental care for the patients with childhood cancer and survivors - A short review | 2020 | European Journal of Molecular and Clinical Medicine | Not associated to radiation caries outcome |
| Badzek S et al. | Side effects of radiotherapy in oral cavity: Diagnosis, prevention and treatment guidelines | 2009 | Lijec Vjesn. | Not associated to radiation caries outcome |
| Ballonoff A, Chen C, Raben D | Current radiation therapy management issues in oral cavity cancer | 2006 | Otolaryngol Clin North Am | Not associated to radiation caries outcome |
| Barker BF and Barker GJ | Oral complications and management of radiation therapy to the head and neck | 1990 | Northwest Dentistry | Not accessible |
| Barry JM | The dentist's role in managing oral complications of cancer therapies | 2005 | Dentistry today | Not accessible |
| Beheshti N, Javid N. | Oral tissue and irradiation therapy | 1978 | Refuat Hapeh Vehashinayim | Not accessible |
| Bennett J. | Oral care of cancer patients undergoing head and neck irradiation | 1979 | Dent Hyg (Chic) | Not accessible |
| Berman CL, Jaffin RA | Radiation caries | 1982 | Journal of the American Dental Association | Letter to editor |
| Bisof V and  Rogulj AA | Head and neck irradiation and oral care of the patient | 2018 | Acta Stomatologica Croatica | Abstract |
| Bornstein M, Filippi A, Buser D | Early and late intraoral sequelae after radiotherapy | 2001 | Schweiz Monatsschr Zahnmed | Not accessible |
| Borovskiĭ EV | Thermophysical properties of the hard dental tissues and the calculation of dental enamel fusion by laser radiation regimens | 1983 | Stomatologiia (Mosk) | Non-ionizing radiation |
| Bortolotti F et al. | Relationship between clinical conditions and oral side effects of head and neck radiotherapy: Piloty study | 2019 | Support Care in Cancer | Abstract |
| Braham RL | Intra-oral problems associated with head and neck irradiation for malignant disease in children and adolescents | 1981 | Practitioner | Not accessible |
| Brauer et al. | Effect of sterilization by gamma radiation on nano-mechanical properties of teeth | 2008 | Dental Materials | Not associated to radiation caries |
| Brennan MT, Woo SB, Lockhart PB | Dental treatment planning and management in the patient who has cancer | 2008 | Dent Clin North Am | Not associated to radiation caries outcome |
| Brignardello-Petersen R | Systematic review suggests important variability in the incidence of caries after radiotherapy | 2020 | J Am Dent Assoc | Commentary |
| Britt, M. R | A role for dentistry in managing the oro-nasopharyngeal irradiated cancer patient | 1981 | Henry Ford Hosp Med J | Not associated to radiation caries outcome |
| Brown, G. M | Management of intraoral problems in the irradiated head and neck cancer patient | 1974 | ALA.J.MED.SCI | Not accessible |
| Bruno JS et al. | Characterization of oral and radiation-related caries microbiota through metagenomic analysis: Preliminary results | 2019 | Support Care in Cancer | Abstract |
| Cai et al. | Raman spectroscopy investigation of human teeth with radiation therapy | 2013 | International Journal of Oral and Maxillofacial Surgery | Abstract |
| Cabrerizo Merino MC | Dental anomalies caused by oncological treatment: case report | 1998 | J Clin Pediatr Dent | Not associated to radiation caries outcome |
| Cacchillo D et al. | Late effects of head and neck radiation therapy and patient/dentist compliance with recommended dental care | 1993 | Spec Care Dentist | Not associated to radiation caries outcome |
| Cai JY et al. | Raman spectroscopy investigation of human teeth with radiation therapy | 2013 | International Journal of Oral and Maxillofacial Surgery | Abstract |
| Calman FM, Langdon J. | Oral complications of cancer | 1991 | British Medical Journal | Editorial |
| Campos Velo MMA | Gamma radiation increases the risk of radiation-related root dental caries | 2017 | Oral Oncology | Letter to editor |
| Carl W. | Oral and dental care for the irradiated patient | 1974 | Quintessence Int Dent Dig | Not accessible |
| Carl W. | Dental management of head and neck cancer patients | 1980 | J Surg Oncol | Not associated to radiation caries outcome |
| Carl W. | Oral and dental care for cancer patients receiving radiation and chemotherapy | 1982 | Quintessence Int Dent Dig | Not accessible |
| Carl W. | Oral complications in cancer patients | 1983 | Am Fam Physician | Not accessible |
| Carl W. | Oral complications of local and systemic cancer treatment | 1995 |  | Not associated to radiation caries outcome |
| Catalano E et al. | Complicanze dentarie post-attiniche: l'interesse radioterapico della sindrome di Gougerot-Sjögren [Postactinic dental complications: radiotherapy involvement in Gougerot-Sjögren syndrome] | 1986 | Minerva Stomatol | Not accessible |
| Chang DT, Sandow PL | Commentary: How radiation damages teeth: Getting to the root of the problem | 2011 | Pract Radiat Oncol | Commentary |
| Chen, Amy Y and Myers, Jeffrey N. | Cancer of the oral cavity | 2000 | Current problems in surgery | Not associated to radiation caries outcome |
| Chua, D. T. T.; Tian, Y; Wei, W. I. | Late oral complications following radiotherapy for head and neck cancers | 2007 | Expert Review of Anticancer Therapy | Not associated to radiation caries outcome |
| De Moor R. | Influence directe et indirecte de la médication (chimiothérapie y comprise) et de l'irradiation sur la pulpe [Direct and indirect effects of medication (including chemotherapy) and irradiation on the pulp] | 2000 | Rev Belge Med Dent | Not associated to radiation caries |
| Declerck D | Soins dentaires chez les patients soumis à une radiothérapie de la tête et du cou [Dental care in patients undergoing radiotherapy of the head and neck] | 1995 | Rev Belge Med Dent (1984) | Not accessible |
| Duarte, V. M.; Tajima, T.; Nabili, V.; Wang, M. B | Dental health of patients receiving intensity modulated radiation therapy vs conventional radiation therapy | 2012 | Otolaryngology - Head and Neck Surgery | Abstract |
| Edgar WM, Bowen WH, Cole MF. | Protein components in saliva and plaque fluid from irradiated primates | 1982 | J Oral Pathol | Not associated to radiation caries |
| Elfenbaum A | Teeth within the irradiation beam | 1970 | Dent Dig. | Not accessible |
| Epstein JB, Barasch A. | Oral and Dental Health in Head and Neck Cancer Patients | 2018 | Cancer Treat Res | Chapter |
| ERSHOFF BH, BAVETTA LA | Potentiating effects of prenatal x-irradiation on dental caries in the rat. | 1958 | Proc Soc Exp Biol Med | Comentary |
| Faria KM | Micromorphology of the Dental Pulp Is Highly Preserved in Cancer Patients Who Underwent Headand Neck Radiotherapy | 2014 | J Endod. | Not associated to radiation caries outcome |
| Ferreira E and  Antunes HS | Importance of adherence to dental treatment in the improvement of oral health after head and neck radiotherapy: case report | 2018 | Support Care in Cancer | Abstract |
| Ferreira E.M et al. | The effect of radiotherapy on the primary deciduos enamel: Analyses μ-EDXRF and FT-Raman | 2013 | Support Care Cancer | Abstract |
| Fretwell DL | The head and neck irradiated patient: dental considerations | 1977 | Va Dent J | Not accessible |
| Galvão-Moreira LV, da Cruz MC | Dental demineralization, radiation caries and oral microbiota in patients with head and neck cancer | 2015 | Oral Oncol. | Commentary |
| Ghalichebaf M, DeBiase CB, Stookey GK. | A new technique for the fabrication of fluoride carriers in patients receiving radiotherapy to the head and neck | 1994 | Compendium. | Not accessible |
| Gilmore ND | Fluoride for the Head and Neck Radiation Patient | 1989 | Mil Med | Not accessible |
| Grötz KA; | Dental care for patients with antineoplastic radiotherapy of the head and neck | 2003 | Strahlenther Onkol | Not accessible |
| Haag, R | An irradiated patient | 1978 | L" Information dentaire | Not accessible |
| Hedge et al. | Protection of wear resistance behaviour of enamel against electron beam irradiation | 2019 | British Dental Journal | Non-ionizing radiation |
| Iancu et al. | RADIOTHERAPY INDUCED TOXICITY FOR OROPHARYNGEAL CANCER PATIENTS: IMPLICATIONS FOR ORAL HEALTH | 2019 | Romanian Journal of Oral Rehabilitation | Not associated to radiation caries outcome |
| Joyston-Bechal, S. | Prevention of dental diseases following radiotherapy and chemotherapy | 1992 | Int Dent J | Not accessible |
| Karcher, H.; Schmid, A. P. | Radiation side effects in the face | 1985 | Rontgenpraxis | Not accessible |
| Katz, S | CHLORHEXIDINE AND RADIATION CARIES - REPLY | 1982 | Journal of the American Dental Association | Commentary |
| Kaufmann M | Zur zahnärztlichen Betreuung von Patienten vor, während und nach Strahlentherapie im Kopf-Hals-Bereich [Patient dental care before, during and after radiation therapy in the head-neck area] | 1980 | SSO Schweiz Monatsschr Zahnheilkd | Not accessible |
| Keys, H. M.; McCasland, J. P.; | Techniques and results of a comprehensive dental care program in head and neck cancer patients | 1976 | Int J Radiat Oncol Biol Phys | Not associated to radiation caries outcome |
| Khanal, B.; Baliga, M.; Uppal, N | Topically applied honey for radiation-induced mucositis: how real is the risk of radiation-related caries? | 2011 | International Journal of Oral and Maxillofacial Surgery | Letter to editor |
| Kochurova, E. V.; Mukhanov, A. A. | Local complications of radiation and chemotherapy treatment of patients with squamous cell carcinoma of the mucosa of the oral cavity | 2018 | Voprosy Onkologii | Not accessible |
| Kozlova AV | Vozmozhnye posledstviia povrezhdeniĭ organov i tkaneĭ pri luchevoĭ terapii zlokachestvennykh opukholeĭ [Possible sequelae of organ and tissue damage during radiotherapy of malignant neoplasms] | 1977 | Med Radiol (Mosk) | Not accessible |
| Leenstra TS | Radio- en/of chemotherapie in het hoofd-halsgebied [Oral aspects of radiotherapy and/or chemotherapy in the head and neck area] | 1990 | Ned Tijdschr Tandheelkd | Not accessible |
| LeMasney NJ et al. | Effects of antimalignancy therapy as seen in the oral cavity of children and young adults--a review | 1990 | J Ir Dent Assoc | Not accessible |
| Levi LE | Dental Treatment Planning for the Patient with Oral Cancer | 2018 | Dent Clin North Am | Not associated to radiation caries outcome |
| Lo, O et al. | Challenging the gold standard of daily fluoride tray use for the prevention of caries in H&N radiotherapy patients | 2012 | Support Care in Cancer | Abstract |
| Longo DL | Late effects from radiation therapy: the hits just keep on coming | 2009 | J Natl Cancer Inst | Editorial |
| Ludwig E. | ahnärztliche Betreuung bei Tumortherapie der Kopf-Hals-Region[nl]- Teil 1: Radiatio [Dental care in case of head and neck cancer--Part I: Radiotherapy]. Laryngorhinootologie | 2008 |  | Not associated to radiation caries |
| Luka, B. et al. | Analyzing oral sequelae of chemo-radio-therapy in the head-neck-region | 2019 | Oncology Research and Treatment | Abstract |
| Madhan, B.; Arunprasad, G.; Krishnan, B.; | Late effects of antineoplastic therapy on the developing dentofacial complex | 2014 | BMJ Case reports | Imagens |
| Makkonen TA, Edelman L, Forsten L | Salivary flow and caries prevention in patients receiving radiotherapy | 1986 | Proc Finn Dent Soc | Not accessible |
| Markwell BD | A case for a dental surgeon at regional radiotherapy centres' | 1992 | Br Dent J | Commentary |
| Marmesat, B et al. | Management of oral problems associated with radiochemotherapy in patients with head and neck cancer | 2016 | International Journal of Clinical Pharmacy | Abstract |
| Martínez, R | Late toxicity and health-related quality of life in locally advanced head and neck cancer treated with RCT after ICT | 2012 | Radiotherapy and Oncology | Abstract |
| Master SB, Sahukar SK, Fernandez VC. | Dental management of the irradiated patient | 1986 | Indian J Cancer | Not accessible |
| Maxymiw WG, Wood RE. | he role of dentistry in head and neck radiation therapy. | 1989 | J Can Dent Assoc | Not associated to radiation caries outcome |
| McCasland J.P. | Dental considerations in head and neck patients: Management of dental and jaw problems | 1975 | EXCERPTA MEDICA | Not accessible |
| McCaul L.K., Hepples M., McCaul J.A. | Dietitian awareness of radiation caries risk and prescribing pattern of oral dietary supplements in head and neck cancer patients | 2012 | European Archives of Oto-Rhino-Laryngology | Abstract |
| McGaw WT, Main JH | Dental care for cancer patients | 1983 | J Can Dent Assoc. | Not accessible |
| Michelet M | Caries and periodontal disease in cancer survivors | 2012 | Evidence based Dentistry | Commentary |
| Murdoch-Kinch CA, Zwetchkenbaum S. | Dental management of the head & neck cancer patient treated with radiation therapy | 2011 | Todays FDA | Not accessible |
| Myers RE, Mitchell DL | Fluoride for the head and neck radiation patient | 1988 | Mil Med | Not accessible |
| Neves, L. V. F. | Feasibility Of Prediction Of Radiation-Related Caries In Head-Neck Cancer Patients Using Machine Learning And Radiomics Features | 2020 | International Journal of Radiation Oncology Biology Physics | Abstract |
| Nguyen, A. M | Dental management of patients who receive chemo- and radiation therapy | 1992 | Gen Dent | Not accessible |
| Oladega, A | Development of guideline for dental care in head and neck cancer patients prior to radiation therapy | 2021 | Radiotherapy and Oncology | Abstract |
| Olczak-Kowalczyk D et al. | The status of dentition and oral hygiene in children after anticancer treatment | 2004 | Annales Academiae Medicae Gedanensis | Not accessible |
| Papadopoulos C | Dental management of the head and neck irradiated patient. | 1991 | Conn Dent Stud J | Not accessible |
| Petkowicz, B | Oral complications after radiotherapy | 2012 | Gastroenterologia Polska | Not accessible |
| Pischetola, S et al. | Dental management of the patient treated for oral cancer: Prevention and rehabilitation | 2021 | Dental Cadmos | Not associated to radiation caries |
| Pochanugool L. | [Oral complications of radiotherapy] | 1984 | J Dent Assoc Thai | Not accessible |
| Pöyry S, Rytömaa I | Kokemuksia sädehoitoa saaneiden potilaiden karieshoidosta [Experience in dental care of the irradiated patients] | 1991 | Suom Hammaslaakarilehti | Not accessible |
| Proietti G et al. | Azione inibitoria svolta dalle radiazioni visibili sulla carie sperimentale del ratto [Inhibitory action of visible radiations on experimental caries in rats]. | 1974 | Ann Ist Super Sanita | Not accessible |
| Reynolds WR | Dental management of the patient receiving radiation therapy for head and neck cancer | 1982 | SCADA J. | Not accessible |
| Ritchie JR et al. | Dental care for the irradiated cancer patient | 1985 | Quintessence Int | Not accessible |
| Ritwik P | Dental Care for Patients With Childhood Cancers | 2018 | Ochsner J | Not associated to radiation caries outcome |
| Roeters FJ, Burgersdijk RC | Een patiënt met afwijkingen in het oro-faciale gebied na stralings-therapie op jeugdige leeftijd [A patient with abnormalities in the oro-facial region following radiotherapy at an early age] | 1981 | Tijdschr Kindergeneeskd | Not accessible |
| Rothstein JP | Radiation therapy and oral care. | 2005 | Dent Today | Not accessible |
| Rubin, R. L.; Doku, H. C. | Therapeutic radiology—the modalities and their effects on oral tissues | 1976 | J Am Dent Assoc | Not associated to radiation caries outcome |
| Sakiko, S et al. | Management of dental caries for prevention of osteoradionecrosis in patients with head and neck cancers | 2017 | Head and Neck | Abstract |
| Schaaf JE | Dealing with oral complications in irradiated cancer patients | 1982 | Dent Indiana Univ | Not accessible |
| Sennhenn-Kirchner S | Dental therapy before and after radiotherapy--an evaluation on patients with head and neck malignancies | 2009 | Clin Oral Investig | Assessed dental care pre-radiotherapy |
| Sever, E. K | Direct radiotherapy induced effects on dental hard tissue | 2021 | Acta Stomatologica Croatica | Abstract |
| Sieńko, E | Oral complications in patients after radiotherapy for head and neck cancer | 2007 | Onkologia Polska | Not accessible |
| Silverman Jr | Radiation therapy of oral carcinoma. I. Effects on oral tissues and management of the periodontium | 1965 | Journal of periodontology | Not associated to radiation caries outcome |
| Simon, A. R.; Roberts, M. W | Management of oral complications associated with cancer therapy in pediatric patients | 1991 | Journal of Dentistry for Children | Not associated to radiation caries outcome |
| Skaleric U, Vrbic V, Budihna M | Postiradiacijske poskodbe v ustni votlini [Postirradiation injuries in the oral cavity] | 1977 | Zobozdrav Vestn | Not accessible |
| Specht L | Oral complications in the head and neck radiation patient. Introduction and scope of the problem. | 2002 | Support Care Cancer | Not associated to radiation caries outcome |
| Stern, S.; Porter, R. W. J | The creation of a preventitive oral hygiene regime for patients following radiation therapy and an audit of its use | 2011 | Oral oncology | Abstract |
| Stover DW | Dental management of head and neck cancer | 1981 | J Md State Dent Assoc | Not accessible |
| Strickland P | Complications of radiotherapy. | 1980 | Br J Hosp Med | Not accessible |
| Sullivan MD, Fleming TJ | Oral care for the radiotherapy-treated head and neck cancer patient | 1986 | Dent Hyg (Chic) | Not accessible |
| Sundara Murthy, S. P. K.; Mahathi; Suresh | Incidence of oral complaints following radiotherapy for treatment of oral cancer | 2020 | International Journal of Research in Pharmaceutical Sciences | Not associated to radiation caries outcome |
| Szpirglas H, Tsamis J, Piade R | Prophylaxie des complications bucco-dentaires de l'irradiation des tumeurs cervico-faciales par la fluoruration topique [Prophylaxis of oral complications of irradiation of cervico-facial tumors by topical fluoride] | 1979 | Inf Dent | Not accessible |
| Szyszkowska, A | Dental care of patients undergoing chemo- and radiotherapy | 2015 | Wspolczesna Onkologia | Not associated to radiation caries outcome |
| Trowbridge JE, Carl W | Oral care of the patient having head and neck irradiation | 1975 | Am J Nurs | Not accessible |
| Tuominen S | Pään ja kaulan alueelle sädehoitoa saavan potilaan suun ja hampaiston hoito [Dental and oral health care of the irradiated patient] | 1991 | Suom Hammaslaakarilehti | Not accessible |
| Vérain A. | La fluorothérapie post-radique [Post-radiation fluorotherapy] | 2004 | Rev Stomatol Chir Maxillofac. | Note |
| Visch LL. | Preventieve mond- en tandzorg bij bestralingspatiënten [Preventive oral and dental care in radiotherapy patients] | 1994 | Ned Tijdschr Tandheelkd | Not accessible |
| Watson, E | Clinical practice guidelines for dental management prior to radiation for head and neck cancer | 2021 | Oral Oncol | Assessed dental care pre-radiotherapy |
| Yen PK | Oral complications of cancer treatment | 2004 | Geriatric Nursing | Not associated to radiation caries outcome |
| Zhang, Y et al. | Late complications and quality of life evaluation after radiation therapy for long-term survivors of nasopharyngeal carcinoma | 2015 | International Journal of Radiation Oncology Biology Physics | Abstract |
| Zhukovskaya, E. | Radioactive caries in patients obtaining therapy for malignant neoplasms | 2019 | Support Care Cancer | Abstract |
