# Supplementary material for: Over 300 Radiation Caries Papers: Reflections From the Rearview Mirror
Source: Front Oral Health. 2022 Jul 14;3:961594. doi: 10.3389/froh.2022.961594 (PMC9330023; doi:10.3389/froh.2022.961594)
Supplement: Supplementary file 3 [file Table_3.docx]

| **Supplementary Table 3.** Methodological approach of the studies included in the RC field (n=348). | | |
| --- | --- | --- |
|  | **N** | **%** |
| Case report | 18 | 5.17 |
| Case series | 4 | 1.14 |
| Case-control | 2 | 0.57 |
| Clinical trial | 12 | 3.44 |
| Cohort | 43 | 12.35 |
| Commentary | 1 | 0.28 |
| Cross-sectional | 36 | 10.34 |
| Narrative review | 105 | 30.17 |
| Preclinical | 114 | 32.75 |
| Quasi-experimental | 3 | 0.86 |
| Systematic review and meta-analysis | 10 | 2.87 |

N=number
